# Effects of Donepezil Hydrochloride on Neuronal Response of Pyramidal Neurons of the CA1 Hippocampus in Rat Model of Alzheimer’s Disease

**DOI:** 10.32598/bcn.9.10.305

**Published:** 2019-03-01

**Authors:** Azade Eskandary, Ahmad Ali Moazedi, Hosein Najaph Zade, Mohamad Reza Akhond

**Affiliations:** 1. Department of Biology, Faculty of Sciences, Shahid Chamran University of Ahvaz, Ahvaz, Iran.; 2. Department of Basic Sciences, Faculty of Veterinary Medicine, Shahid Chamran University of Ahvaz, Ahvaz, Iran.; 3. Department of Statistics, Faculty of Mathematical Sciences and Computer, Shahid Chamran University of Ahvaz, Ahvaz, Iran.

**Keywords:** Alzheimer’s Disease, Electrophysiology, Donepezil, Rats

## Abstract

**Introduction:**

Donepezil (DON), an Acetylcholinesterase Inhibitor (AChEI), is widely used in the treatment of Alzheimer’s Disease (AD). The current study aimed at evaluating the effect of donepezil hydrochloride on pyramidal neuron response in CA1 region of a rat model of AD.

**Methods:**

In the current experimental study, adult male Wistar rats were randomly divided into four groups: Nucleus Basalis Magnocellularis (NBM) lesion (the lesions were induced by an electrical method of 0.5 m A, for 3 s in NBM) and three donepezil groups (lesions plus 5, 10, and 15 mg/kg donepezil intraperitoneal injection). Neuronal spontaneous activity to injection of the donepezil and saline were recorded in CA1 region of hippocampal.

**Results:**

The obtained results showed that IntraPeritoneal (IP) injection of donepezil (10 and 15 mg/kg) increased neuronal spontaneous activity in the rat model of AD.

**Conclusion:**

The current study results suggested that acute IP injection of donepezil increased neuronal response in CA1 region of hippocampal in a rat model of AD.

## Highlights

- Alzheimer’s disease model was induced by creating lesions in Nucleus Basalis Magnocellularis (NBM).
- The spontaneous-activity of the pyramidal neuron of the CA1 area of the hippocampus in the rat model of AD was investigated.
- Decreasing the spontaneous activity of pyramidal neurons destructed NBM.
- Donepezil administration improved the activity of pyramidal neurons.

## Plain Language Summary

Alzheimer’s Disease (AD) is one of the most common diseases of old age. Based on studies, the level of neurotransmitter acetylcholine of the brain reduces due to AD. Cholinergic system in the brain is related to learning and memory processes. In our study, the Nucleus Basalis Magnocellularis (NBM), a cholinergic source in the rat brain, was destroyed and a model of animal AD was created. Donepezil increases acetylcholine levels by inhibiting acetyl-cholinesterase enzyme in the brain. In the current study, with the destruction of NBM, the level of spontaneous activity of the pyramidal neuron of hippocampal, one of the important centers of learning and memory, reduced but donepezil injection improved the activity of these neurons.

## 1. Introduction

Azheimer Disease (AD) is a progressive and irreversible neurodegenerative disease characterized by a decline in cognitive function, behavior disturbance, and impairment in daily functions (Umont & Beal, 2011). Currently, around 35 million people have AD, which ends in death within 3 to 9 years after diagnosis (Noetzli & Eap, 2013; Zhang, Chen, An, & Wang, 2016). AD is related to the degeneration of cortical projections of the cholinergic neurons from the basal forebrain. There is also a strong correlation between neuronal decline and cortical cholinergic deficiency and degree of cognitive deficits (Grothe et al., 2014).

Nucleus Basalis Magnocellularis (NBM) is one of the basal forebrain structures that 90% of its neurons are cholinergic. In patients with AD, 50% to 80% of cholinergic neurons in the Nucleus Basalis (NB) that project to the hippocampus and cortex are depleted (Whitehouse, Price, Clark, Coyle, & DeLong, 1981). Since NBM in rodents is equivalent to the nucleus basalis of Meynert in humans, NBM-lesion rats can be used as an animal model of AD (Cutuli et al., 2009). Studies show that in NBM-lesion rat, brain cholinergic markers such as release of acetylcholine, and choline acetyltransferase activity reduce and learning and memory are destroyed (Wu et al., 2005; Meyer et al., 1987).

Acetylcholinesterase Inhibitors (AChEI), which prohibit the enzymatic depreciation of acetylcholine in the synaptic cleft, are the first-line treatment of AD (Easton, et al., 2013). Donepezil, the most commonly prescribed drug for AD, entered the market as a remedy for mild-to-moderate AD in 1996. Due to the centrally irreversible action of AChE inhibitors, donepezil impresses the amelioration of cognitive functions in humans (Ginani, et al., 2011). Besides, it prohibits β-Amyloid Precursor Protein (APP) processing (Kimura, Akasofu, Ogura, & Sawada, 2005).

Regulates voltage-gated calcium and potassium channels, and up-regulates nAChRs in cortex (Kume et al., 2005). In addition, different types of receptors, including N-Methyl-D-Aspartate (NMDA) (Moriguch, Shioda, Han, Yeh, & Narahashi, 2005), ϭ1 (Meuneir, Ieni & Maurice, 2006), and α4nACh (Akaika, Takada-Takatori, Kume, & Izumi, 2010) are the potential sites of interaction for donepezil that may contribute to its therapeutic effects. Donepezil is characterized by the protection of neurons from Aβ-induced neurodegeneration (Arias, Gallego-Sandin, Villarroya, Garcia, & Lopez, 2005).

Extracellular single unit recording methods are used to obtain valuable information about the structural features of the Central Nervous System (CNS). Additionally, they are recently used to study the behavioral patterns of neural discharging of the CNS (Donald, Humphrey & Schmid, 1990). Most studies on AD consider the cognitive and memory deficits, and spontaneous activity of neurons in the brain is less investigated. Therefore, in the current study, the effect of donepezil on electrical firing of CA1 pyramidal neurons was evaluated using in vivo single-unit recording method in rats.

## 2. Methods

### 2.1. Study animals

Adult male Wistar albino rats (weight: 220±20 g) were purchased from animal house of Ahvaz Jundishapur University of Medical Science (AJUMS). Animals were kept in cages each of three at controlled room temperature of 22±2°C, and 12:12 hour light/dark cycle (light from 07:00 AM) was maintained. Animals had free access to water and food. All experiments were performed according to the instructions for the care and maintenance of laboratory animals.

### 2.2. Study drugs

Donepezil hydrochloride (Sigma-Aldridge), dissolved in sterile saline (0.9%) as a vehicle, was used in the current study. Control group animals received saline. The drug was prepared immediately before injection and administered in a volume of 1 mL/kg.

### 2.3. Experimental procedure

In the current study, extracellular single-unit recording from hippocampal pyramidal neuron was conducted in the anesthetized animal. Testing session was performed in a quiet room, at the room temperature of 25±1°C. A total of 40 male Wistar rats were tested in the experiments. There were four groups (n=10) as follow: 1. Lesion group in which the NBM of animals was destroyed bilaterally with electrical current+saline; 2. NBM lesion plus donepezil 5 mg/kg; 3. NBM lesion plus donepezil 10 mg/kg; and 4. NBM lesion plus donepezil 15 mg/kg.

Recovery period for lesion group was one week. After the recovery period, they were prepared for single-unit recording, i.e. after baseline recording (15 minutes), donepezil or saline IP was injected and the recording was continued for 105 minutes afterward. Change in firing activity was calculated from the recorded neuron after the drug injection and interpreted as an indicator of the impression of the drug on the electrical features of neurons.

### 2.4. Induced Alzheimer’s model

**Figure 1.**
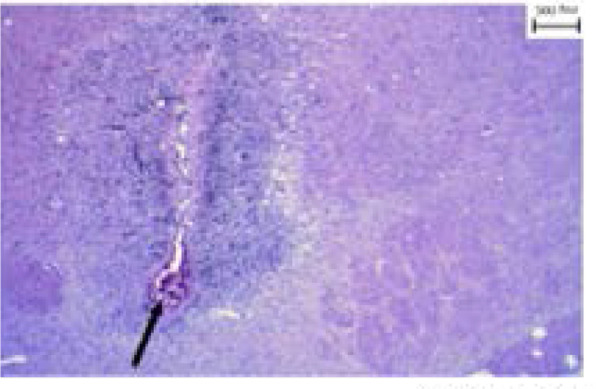
Electrical damage site in the NBM

To create the AD model, the animals were first subjected to anesthesia with ketamine (78 mg/kg, IP, Alfasan, Holland) and xylacin (3 mg/kg, IP, Alfasan, Holland), and then the NBM of animals was destroyed bilaterally with electrical current (0.5 mA for 3 s) according to the stereotactic coordinates (AP −1.3 mm, ML ±2.8 mm, DV −7.6 mm) (Rabiei, Rafieian-kopaei, Heidarian, Saghaei, Mokhtari, 2014). One week after surgery, the animals were prepared for the experiment of electrophysiology and single-unit recording. A histological sample confirming the degradation of the NBM of AD was presented (Figure 1).

### 2.5. Animal preparation and stereotactic surgery

The administration of ketamine for anesthesia blocks NMDA receptors, which leads to a change in electro-physiological record. Therefore, to anesthetize the animals a substance should be administered that does not block the brain receptors. Urethane is a suitable material for this purpose. Animals were deeply anesthetized with urethane (1.5 g/kg, IP; Sigma-Aldrich, Germany) with supplemental doses (0.1 g/kg) every one hour as needed to maintain a deep and stable level of anesthesia as determined by lack of movement in response to a strong tail pinch.

To diminish the respiratory efforts and keep a stable airway pending on the recording, the rats underwent tracheostomy surgery. Briefly, the hair front of the neck was shaved and a cut was created. Then the muscles and smooth tissue of the neck were removed to the trachea. A gap was created in the trachea and a polyethylene tube inserted into the lower part of the trachea and tightened with sutures. Then, the animal was softly placed in a stereotactic device (Stoelting, USA).

The skin of the rats was cleared to reveal the cranial surface, and Bregma was designated as the reference point for stereotactic. A 2-mm diameter hole was created at the top of the CA1 area (AP −3.8 mm, ML ±2.2 mm, DV −2.4 mm) of hippocampus. The body temperature was maintained for the entire experiment at 36–37°C with a heating pad.

### 2.6. Extracellular single-unit recording and data acquisition

Extracellular recording from individual neuron were performed with tungsten microelectrode (parylene coated, shaft diameter 127 μm, tip impedance 5 MΩ, Harvard apparatus). Microelectrode was stereotactically advanced into the CA1 of hippocampus. Thereafter the electrode was moved slowly in the layer of pyramidal neurons using a microelectrode driver till a specific spike activity is recorded with a signal-to-noise ratio of >2 separation of the background activity.

Spike signals were amplified (×10000 gain; 300 Hz, and 10 kHz for low and high filters, respectively) and displayed continuously on a storage oscilloscope as signals. The spike frequency was calculated and transmitted online in time bins of 1000 ms for the entire recording time by online sorter software (Spike; Science Beam, Tehran, Iran). The action potentials of the baseline activity were separated using a windows discriminator, which produced output pulses for single-units based on the spike height, which calculated the number of spikes per unit time. In this experiment, recording time for data gathering was 7200 s with bin size 1000 ms constantly stored on the hard disk and average frequency was computed by computer (Green & Arenos, 2007).

Pyramidal neurons of the hippocampus were acquainted based on spontaneous frequency of eight spikes or less (Green & Arenos, 2007). Since a pyramidal neuron with permanent firing frequency and constant spike amplitude and wave forms was detected as a baseline, the recording was continued for about 15 minutes. Then the drug was injected and the recording was carried on for about 105 minutes. In the current study, discharge of each neuron was calculated at a time interval of 60 s bins using a data acquisition program to create a Peri-Stimulus Time Histograms (PSTHs) with a time interval of 15 minutes before the injection to 105 minutes after the injection of the drug or vehicle.

The data were analyzed off-line using the home analysis software for windows. In order to detect the neuronal response patterns to saline, donepezil 5, 10, and 15 mg/kg administration, the total period of perception was cut into 60-s time bins. Increase or decreases of the activity of neurons as two-fold time standard deviation from baseline activity for three consecutive points were considered as an excitatory or inhibitory response.

### 2.7. Histological verification

**Figure 2.**
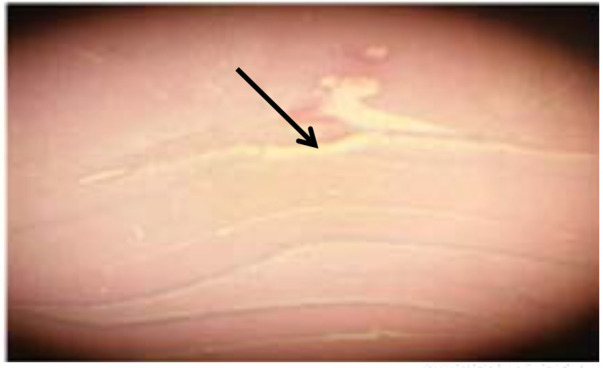
Place of electrophysiological recording in CA1 layer

After the end of the electrophysiological record, the animals’ brains were removed and fixed in formalin 10%. After blocking, 20-μm sections were taken from the locations near the electrode. The sections were stained with Hematoxylin and Eosin (H&E). Then, a microscope (Japan; Olympus EX51) was used to determine the location of recording in the CA1 region of the hippocampus (Figure 2).

### 2.8. Statistical analysis

Recording was performed before and 105 minutes after IP administration of saline and donepezil. The obtained data were analyzed with SPSS V. 20. The paired sample t test was employed to evaluate drug effect on the neural firing rate before and after drug injection. In addition, Graphpad Prism version 6.07 was used to plot the effect of the drug on the number of stimulatory, inhibitory, and ineffective neurons. The data were presented as Mean ± Standard Error of the Mean (SEM). P<0.05 was considered statistically significant.

## 3. Results

**Figure 3.**
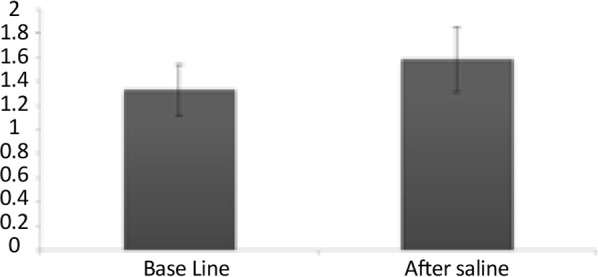
Effect of saline on the firing frequency of the CA1 pyramidal neurons

**Figure 4.**
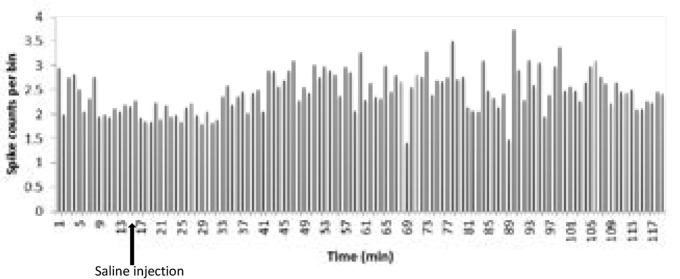
Histogram of firing pattern of CA1 pyramidal neurons before and after saline injection

To investigate the role of saline in the electrical firing of pyramidal neuron in the CA1 of the hippocampus, 0.2 mL saline was injected IP subsequent to the baseline recording. Then the neural firing was recorded for 105 minutes. The paired samples t test of the stimulatory response of neurons in the lesion group to saline injections showed no significant increase in the frequency of the neurons after injection compared with the base activity (t=−1.01, df=15; P>0.05) (Figures 3 and 4).

Fifteen neurons from 10 individual rats were recorded, from which saline excited 3(50%), inhibited 2(50%), and unaffected 10(50%). The onset of stimulation was observed from 47 to 56 minutes after intraperitoneal injection. Also, an investigation of the mean increase in the activity of the pyramidal neurons in the CA1 region of the hippocampus revealed that saline injections increased the activity in three neurons by 25 to 70% and reduced activity in two neurons by 60 to 70%.

**Figure 5.**
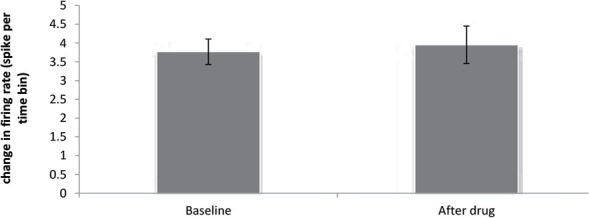
Effect of donepezil 5 mg/kg on the firing frequency in CA1 pyramidal neurons (P>0.05)

**Figure 6.**
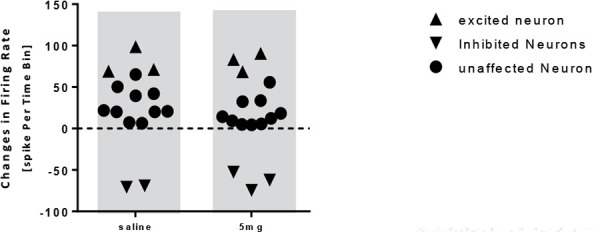
Scatterplot representing the response of pyramidal neurons to saline and donepezil 5 mg/kg injection

In the donepezil 5 mg/kg group, the paired samples t test showed no significant increase in the frequency of the neurons after injection compared to baseline activity (t=−0.416, df=16; P>0.05) (Figure 5). In this group, 16 neurons from 10 individual rats were recorded and it was observed that donepezil 5 mg/kg unaffected 10 neurons, inhibited 3 neurons, and excited 3 ones (Figure 6).

**Figure 7.**
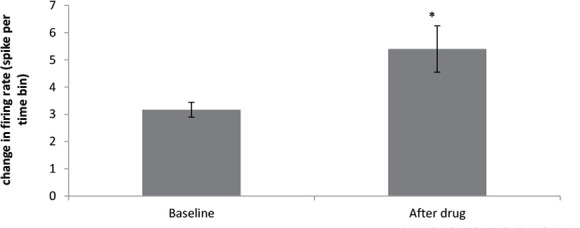
Effect of donepezil 10 mg/kg on the firing frequency in CA1 pyramidal neurons (P<0.05)

**Figure 8.**
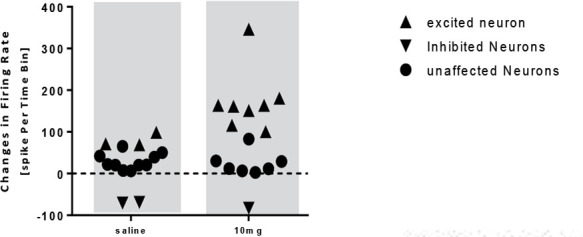
The scatterplot representing the response of pyramidal neurons to saline and donepezil 10 mg/kg injection

The onset of stimulation initiated 47 to 64 minutes after the administration of donepezil 5 mg. In the group receiving donepezil 10 mg/kg, the paired samples t-test showed a significant increase in the firing frequency of the neurons after injection compared with that of the baseline activity (t=−2.663, df=14; P<0.05) (Figure 7). In this group, 14 neurons from 10 rats were recorded and donepezil 10 mg had an excitatory effect on 8 neurons, unaffected 5 neurons, and inhibited 1 neuron (Figure 8).

The onset of the stimulation was observed 47 to 56 minutes after the administration. Also, the administration of donepezil 10 mg resulted in an increased activity of 100 to 115% in 3 neurons, a rise of 160 to 180% in 4 neurons, and an increase in neuronal activity of 346% in 1 neuron, and resulted in an 80% reduction in the neuron activity.

**Figure 9.**
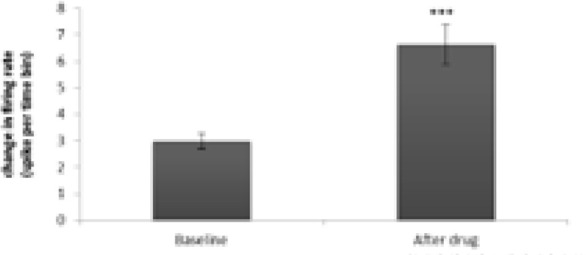
Effect of donepezil 15 mg/kg on firing frequency in CA1 pyramidal neurons (P<0.001)

**Figure 10.**
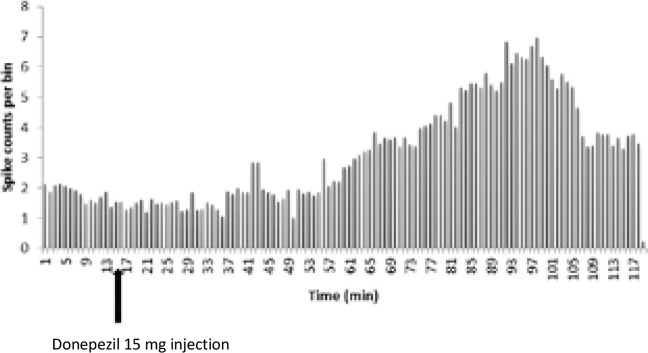
Firing pattern of CA1 pyramidal neuron before and after donepezil 15 mg/kg injection

**Figure 11.**
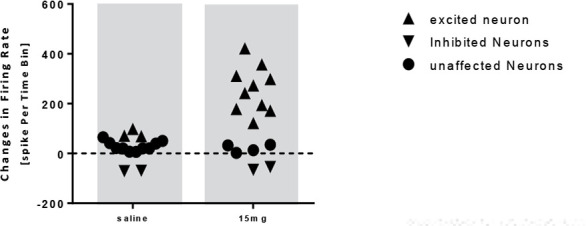
Scatterplot representing the response of pyramidal neurons to saline and donepezil 15 mg/kg injection

The statistical analysis in the 15 mg/kg donepezil group showed a significant increase in the frequency of firing of neurons after injection compared with that of the baseline activity (t=−4.485, df=15; P<0.001) (Figures 9 and 10). In this group, 16 neurons from 10 rats were recorded and it was observed that donepezil 15 mg had an excitatory effect on 10 neurons, unaffected 4 neurons and inhibited 2 neurons (Figure 11). Initiation of the stimulation was observed 42 to 61 minutes after injection. Also, the administration of donepezil 15 mg resulted in an increase of 120 to 190% in 4 neurons, an increase of 240–270% in 3 neurons, and 3 neurons resulted in an increase of 310 to 420% of the neuron activity. Donepezil 15 mg resulted in a 55 to 65% reduction in activity in 2 neurons.

## 4. Discussion

In the current study, saline and donepezil at doses of 5, 10, and 15 mg/kg were injected into the AD model rats, and it was observed that donepezil at doses of 10 and 15 mg/kg had a positive effect and spontaneously increased the activity of the pyramidal neurons in the CA1 region. Cholinergic fibers spread from the medial septum and the basal forebrain to the entire neocortex and hippocampus (Dutar, Bassant, Senut, & Lamour, 1995). The release of acetylcholine and the activation of muscarinic acetylcholine receptors coupled with G-protein promotes learning and memory in a wide range of behavioral tests (Stackman et al., 2002). Thus, the regulation of cholinergic system is one of the treatments of AD. Therefore in man and in laboratory models, acetylcholine release, and mAchR signaling helps the acquisition of new behavior and facilitates learning (Giessel & Sabatini, 2010).

In hippocampal CA1 pyramidal neurons, cholinergic stimulation acts through routine muscarinic receptors and increases the ability to stimulate by regulating synaptic behavior (Buchanan, Petrovic, Chamberlain, Marrion, & Mellor, 2010) and intrinsic properties of the membrane (Park & Spruston, 2012). The activation of muscarinic receptors leads to depolarization and increased stimulation of pyramidal CA1 neurons by inhibiting potassium activity and activating non-specific cationic conductivity (Yamanda-Hanff & Bean, 2013).

According to several studies, acetylcholine release and activation of mAchR increases glutaminergic synaptic responses in the CNS (Giessel & Sabatini, 2013). In the hippocampus, mAchR agonists stimulate Long-Term Potentiation (LTP)-dependent NMDA receptor in the synapses between the pyramidal CA1 and CA3 neurons (Fernandez, Nun, Borde, Malinow & Bun, 2008).

The potentials of glutaminergic synapses and dendritic calcium transfer are regulated by a spectrum of ionic channels of non-glutamate receptors, including potassium, sodium, calcium, and potassium channels sensitive to the concentration of calcium of the SK species (Andrá sfalvy, Makara, Johnston, & Magee, 2008; Bloodgood, Giessel, & Sabatini, 2009).

Many of these ion channels are regulated by mAchR and other receptors coupled with G-protein (Faber et al., 2008) and the blockage of these channels leads to a synaptic deformation (Lin, Luja, Watanabe, Adelman, & Maylie, 2008). It is determined that SK channels interfere with the potential of subsequent hyperpolarization. In the CA1 and CA3 synapses, the block of the K-channel strengthens the synaptic potential and calcium transmission and facilitates LTP induction (Ngo-Anh, et al., 2005). In addition, the in vivo implementation of SK channel antagonists leads to increased acquisition in the hippocampal-dependent behaviors (Giessel & Sabatini, 2010).

Moriguchi et al. (2005) reported that donepezil regulated the activity of NMDA receptor in rat cortical neurons in a primary culture using a patch-clamp technique. It was observed that donepezil stimulated NMDA induction in concentrations of 0.01 to 100 μm in bipolar neurons. Therefore, donepezil was useful in stimulating the cholinergic system by inhibiting acetylcholinesterase and enhancing the activity of the NMDA system and reversing the activity of these two neurotransmitter systems to normal levels in improving learning, memory, and cognition in patients (Moriguchi et al., 2005).

Investigations reveal that injection of Aβ 1–42 peptide at 200 nM concentration did not have a significant effect on the basic characteristics of the spike population of the hippocampal slice, but led to an impairment of LTP induction in the hippocampal slice after HFS. Injection of 1 μm donepezil resulted in a tendency to increase LTP in the hippocampal slice. Co-administration injection of Aβ peptide and donepezil resulted in the elimination of the repressive effect of Aβ peptide on LTP induction. These results emphasize the possible new mechanisms of donepezil therapeutic activity, which includes increased acetylcholine transfer by inhibiting acetylcholinesterase or increasing the number of nicotine receptors and neuronal protection (Kapai & Bukanova, 2012).

Donepezil target molecules, besides acetylcholinesterase, are NMDA receptors, sigma receptors 1, and potassium and calcium voltage channels, which may show beneficial effects of donepezil on LTP through one of these mechanisms (Kapai, Solentseva, & Skrebitskii, 2012). Therefore, according to the results of the current study, as well as reports of other tests, donepezil increased the activity of the pyramidal neurons from the CA1 hippocampal region and can be considered as an effective treatment to reduce AD.

## Ethical Considerations

### Compliance with ethical guidelines

The Ethics Committee of Shahid Chamran University of Ahvaz confirmed the research protocol (Code: EE/97.24.3.17933/scu.ac.ir).
